# Estimation of Maximum Tibial Length From Measured Anthropometric Parameters in a North Indian Population

**DOI:** 10.7759/cureus.116786

**Published:** 2026-09-23

**Authors:** Anju Choudhary, Apurba Patra, Pooja Garg

**Affiliations:** 1 Anatomy, All India Institute of Medical Sciences, Bathinda, Bathinda, IND; 2 Anatomy, Mahatma Gandhi Medical College and Hospital, Jaipur, IND

**Keywords:** anthropometry, forensic anthropology, indian population, regression analysis, stature estimation, tibia length

## Abstract

Background: Accurate tibia length estimation is essential for anthropological, clinical, and forensic applications. However, population-specific data are crucial due to variations in body proportions. This study aims to establish a regression model for estimating the maximum tibial length (MTL) based on easily measurable tibial anthropometric parameters in the Indian population.

Methods: A cross-sectional study was conducted on 90 adult dry tibia bones. Measurements of various tibial anthropometric parameters, including tibial length (proximal to distal points), tibial breadth (medial and lateral condyle widths), and tibial circumferences, were taken using standard osteometric techniques. Data were analyzed using statistical methods, including correlation analysis and multiple regression models, to determine the best predictors of MTL. The sample was stratified by gender to account for sex-specific variations in tibia length.

Results: A total of 90 observations were analyzed. Several variables showed significant deviation from normality. Males were significantly older and had higher values for MTL, bicondylar tibial width (BTW), anterior-posterior intercondylar diameter (APID), anterior-posterior diameter of the medial condyle (APDMC), and transverse diameter of the medial condyle (TDMC) compared to females (all p < 0.01). Correlation analysis demonstrated strong positive associations between MTL and APID (r = 0.643, p < 0.001) and between APID and distal articular surface length (DASL) (r = 0.740, p < 0.001).

Conclusion: The study successfully derived population-specific regression models for estimating MTL from anthropometric measurements in the Indian population. These models can be applied in forensic investigations and anthropological research to estimate stature accurately. However, further validation studies in diverse Indian subpopulations are recommended to enhance the generalizability of the findings.

## Introduction

The tibia is frequently utilized for metric analysis because of its excellent preservation in both forensic and archaeological contexts. In dry skeletal remains, tibial measurements have been applied to assess various components of the biological profile, either as primary or supplementary indicators. The tibia is also amenable to anthropometric assessment in living individuals, as much of its length is readily palpable and accessible for measurement [1].

Stature estimation has long been an important area of research in forensic science and physical anthropology, as stature constitutes a key component of the biological profile used for human identification. Reliable estimates of stature can assist in the identification of individuals involved in mass disasters, mass burials, and forensic investigations, as well as in cases involving missing persons. The established positive relationship between stature and the lengths of the long bones has therefore facilitated the use of long-bone measurements for reconstructing and estimating the stature of unidentified individuals [2-4].

Numerous anthropological studies have emphasized that the accuracy of stature estimation can be improved when regression equations are developed specifically for the population under investigation, owing to inter-population differences in skeletal proportions and body dimensions [5-7].

In the present study, we investigated the morphometric characteristics of the tibia and evaluated selected proximal tibial parameters for their association with maximum tibial length (MTL). Based on these relationships, linear regression equations were developed to estimate MTL, with the aim of providing population-specific equations applicable to selected Indian populations.

## Materials and methods

This research involved a cross-sectional observational study utilizing 90 (58 male and 32 female) well-preserved contemporary adult human tibiae sourced from the Department of Anatomy, All India Institute of Medical Sciences. Osteological specimens were selected using a convenience sampling method based on their availability. Bones selected for the study had completely closed epiphyseal plates, indicating that they belonged to adults. Bones with pathological conditions, deformations, fractures, or putrefied and disintegrated parts were excluded from the study. The study was conducted in full compliance with institutional guidelines for cadaveric research and adhered to the ethical principles outlined in the Declaration of Helsinki and its later amendments, as well as international standards for research involving human anatomical material.

Methods of measurements

Measurements for MTL were taken using an osteometric board. Digital vernier calipers (Mitutoyo, Kawasaki, Japan) with a least count of 0.05 were used to measure 13 morphometric parameters, as shown in Figure 1.

**Figure 1:**
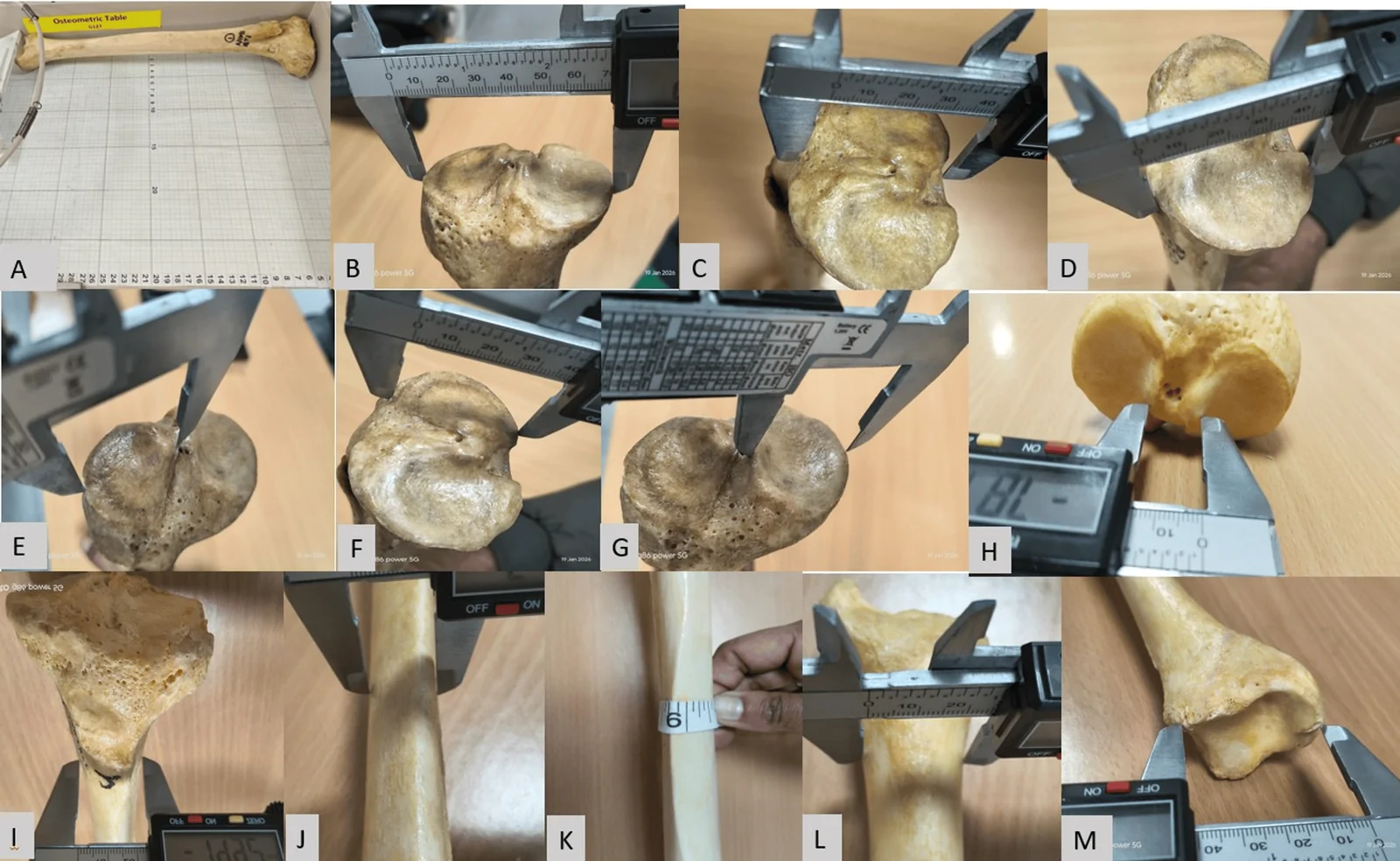
Osteometric measurements of the tibia. Osteometric measurements of the tibia: (A) maximum tibial length (MTL); (B) bicondylar tibial width (BTW); (C) anterior-posterior intercondylar diameter (APID); (D) anterior-posterior diameter of the medial condyle (APDMC); (E) transverse diameter of the medial condyle (TDMC); (F) anterior-posterior diameter of the lateral condyle (APDLC); (G) transverse diameter of the lateral condyle (TDLC); (H) intercondylar distance (ID); (I) proximal shaft diameter (PSD); (J) midshaft transverse diameter (MSTD); (K) midshaft circumference (MSC); (L) distal shaft diameter (DSD); and (M) distal articular surface length (DASL).

Measurements were taken based on a standardized protocol, as described by Martin et al. [8] and Buikstra et al. [9]. The following measurements were taken: maximum tibial length (MTL) is measured as the maximum distance from the highest point of the upper part of the tibia to the lowest point of the tibia using the osteometric board (Figure 1A); bicondylar tibial width (BTW) is measured as the maximum transverse distance from the lateral side of the lateral condyle to the medial side of the medial condyle (Figure 1B); anterior-posterior intercondylar diameter (APID) is measured as the maximum distance from the anterior intercondylar area to the posterior intercondylar area (Figure 1C); anterior-posterior diameter of the medial condyle (APDMC) is measured as the maximum anterior-posterior distance of the medial condyle (Figure 1D); transverse diameter of the medial condyle (TDMC) is measured as the maximum distance from the medial side of the medial condyle to the medial intercondylar tubercle of the intercondylar eminence (Figure 1E); anterior-posterior distance of the lateral condyle (APDLC) is measured as the maximum anterior-posterior distance of the lateral condyle (Figure 1F); the transverse diameter of the lateral condyle (TDLC) is measured as the maximum distance from the lateral side of the lateral condyle to the lateral intercondylar tubercle of the intercondylar eminence (Figure 1G); intercondylar distance (ID) is measured as the maximum distance from the medial intercondylar tubercle to the lateral intercondylar tubercle (Figure 1H); proximal shaft diameter (PSD) is measured as the maximum transverse distance of the proximal part of the shaft just inferior to the tibial tuberosity (Figure 1I); midshaft transverse diameter (MSTD) is measured as the maximum transverse distance of the midpoint of the shaft (Figure 1J); mid-shaft circumference (MSC) is measured as the maximum circumference at the mid-length of the shaft (Figure 1K); distal shaft diameter (DSD) is measured as the maximum transverse distance of the lower end of the shaft (Figure 1L); and distal articular surface length (DASL) is measured as the maximum transverse distance of the inferior surface of the tibia (Figure 1M).

Statistical analysis

Data were analyzed using IBM SPSS Statistics version 29 (IBM Corp., Armonk, NY, USA). Continuous variables are presented as mean ± standard deviation (SD). Pearson's correlation analysis was performed to evaluate the relationship between MTL and anthropometric variables. Data were analyzed separately for males and females to account for sex-specific differences in tibial length. A p-value < 0.05 was considered statistically significant.

## Results

The analysis encompassed 90 valid observations for each variable assessed. Age ranged from 18 to 90 years (approximately), with males being significantly older (p = 0.004) than females.

Independent samples t-tests revealed statistically significant differences between genders across several metrics: males were significantly older (p = 0.004) (Figure 2A) and scored higher on MTL (p < 0.001) (Figure 2B), BTW (p < 0.001) (Figure 2C), and APID (p < 0.001) (Figure 2D).

Additionally, significant differences were noted in APDMC (Figure 2E) and TDMC (Figure 2F), with males consistently scoring higher (Table 1).

**Figure 2:**
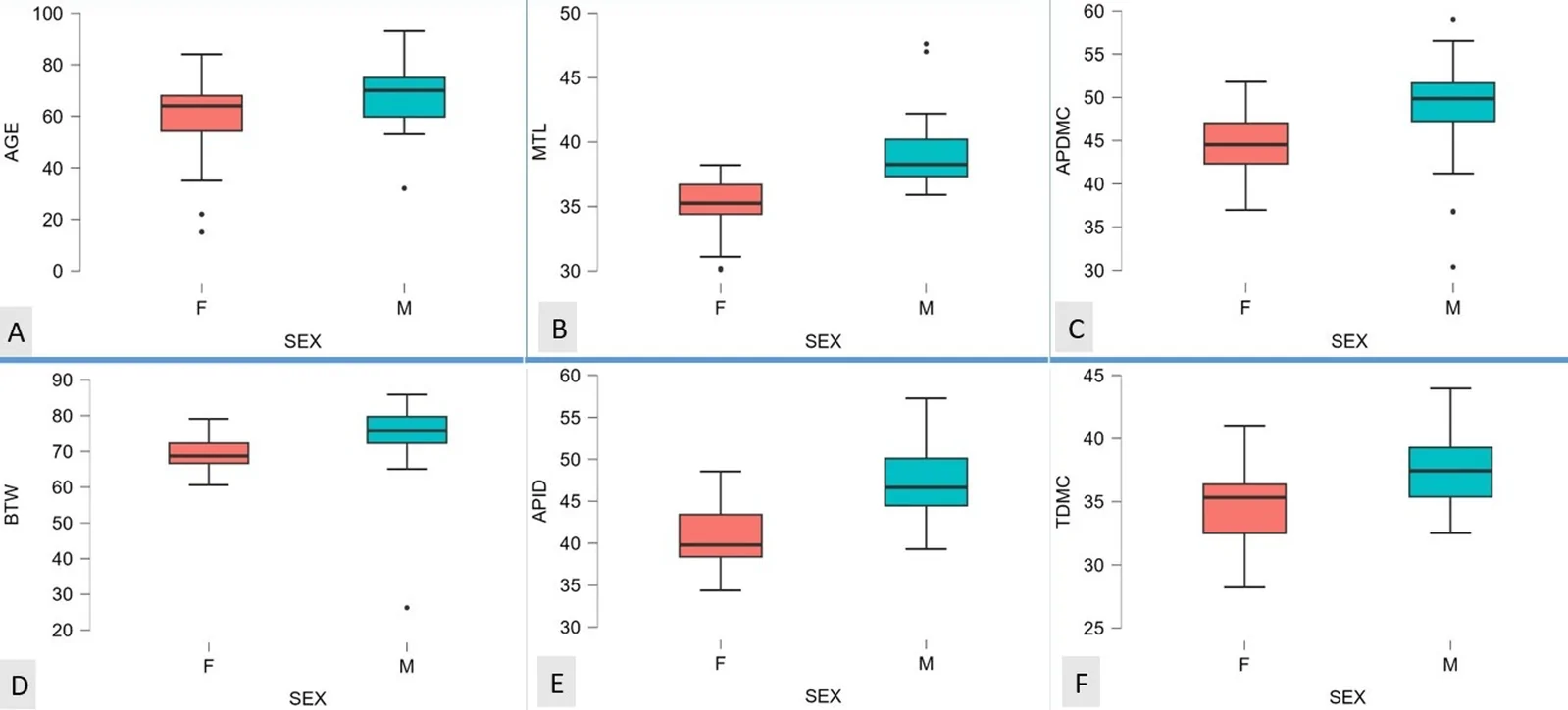
Gender-wise comparison of age and tibial measurements. Independent-samples t-test showing significant differences between males and females in (A) age (years), (B) maximum tibial length (mm), (C) anterior-posterior diameter of the medial condyle (mm), (D) bicondylar tibial width (mm), (E) anterior-posterior intercondylar diameter (mm), and (F) transverse diameter of the medial condyle (mm). p = 0.004 for age and p < 0.001 for all tibial measurements. MTL: maximum tibial length; APDMC: anterior-posterior diameter of the medial condyle; BTW: bicondylar tibial width; APID: anterior-posterior intercondylar diameter; TDMC: transverse diameter of the medial condyle; mm: millimeter; male tibia: 58; female tibia: 32.

**Table 1:** Gender-wise comparison (independent samples t-test). MTL: maximum tibial length; BTW: bicondylar tibial width; APID: anterior-posterior intercondylar diameter; APDMC: anterior-posterior diameter of the medial condyle; TDMC: transverse diameter of the medial condyle. All measurements are in millimeters.

Variable	Female (Mean ± SD) (n = 32)	Male (Mean ± SD) (n = 58)	t-value	p-value	Significance
Age (years)	58.22 ± 18.60	67.67 ± 11.766	2.95	0.004	Significant
MTL (mm)	35.25 ± 2.11	38.25 ± 2.34	6.02	<0.001	Significant
BTW (mm)	68.72 ± 4.23	75.78 ± 8.22	4.53	<0.001	Significant
APID (mm)	39.80 ± 3.34	46.65 ± 3.94	8.32	<0.001	Significant
APDMC (mm)	44.52 ± 3.52	49.85 ± 4.97	5.36	<0.001	Significant
TDMC (mm)	34.83 ± 3.24	37.48 ± 2.71	4.14	<0.001	Significant

Correlation analysis (Table 2) identified several significant relationships, including a strong positive correlation between MTL and APID (r = 0.643, p < 0.001), as well as between APID and DASL (r = 0.740, p < 0.001).

**Table 2:** Pearson’s correlation test results as identified between various measured parameters. MTL: maximum tibial length; APID: anterior-posterior intercondylar diameter; BTW: bicondylar tibial width; DASL: distal articular surface length; ID: intercondylar distance; DSD: distal shaft diameter; MSC: mid-shaft circumference.

Variables compared	r	p-value	Interpretation
MTL-APID	0.643	<0.001	Strong positive
APID-DASL	0.740	<0.001	Strong positive
BTW-ID	0.388	<0.001	Moderate positive
DSD-MSC	-0.227	0.032	Weak negative

Additionally, BTW showed a significant positive correlation with ID (r = 0.388, p < 0.001). Conversely, negative correlations were observed between DSD and several other variables, indicating that higher scores in DSD were associated with lower scores in measures such as MSC (r = -0.227, p = 0.032) (Figure 3).

**Figure 3:**
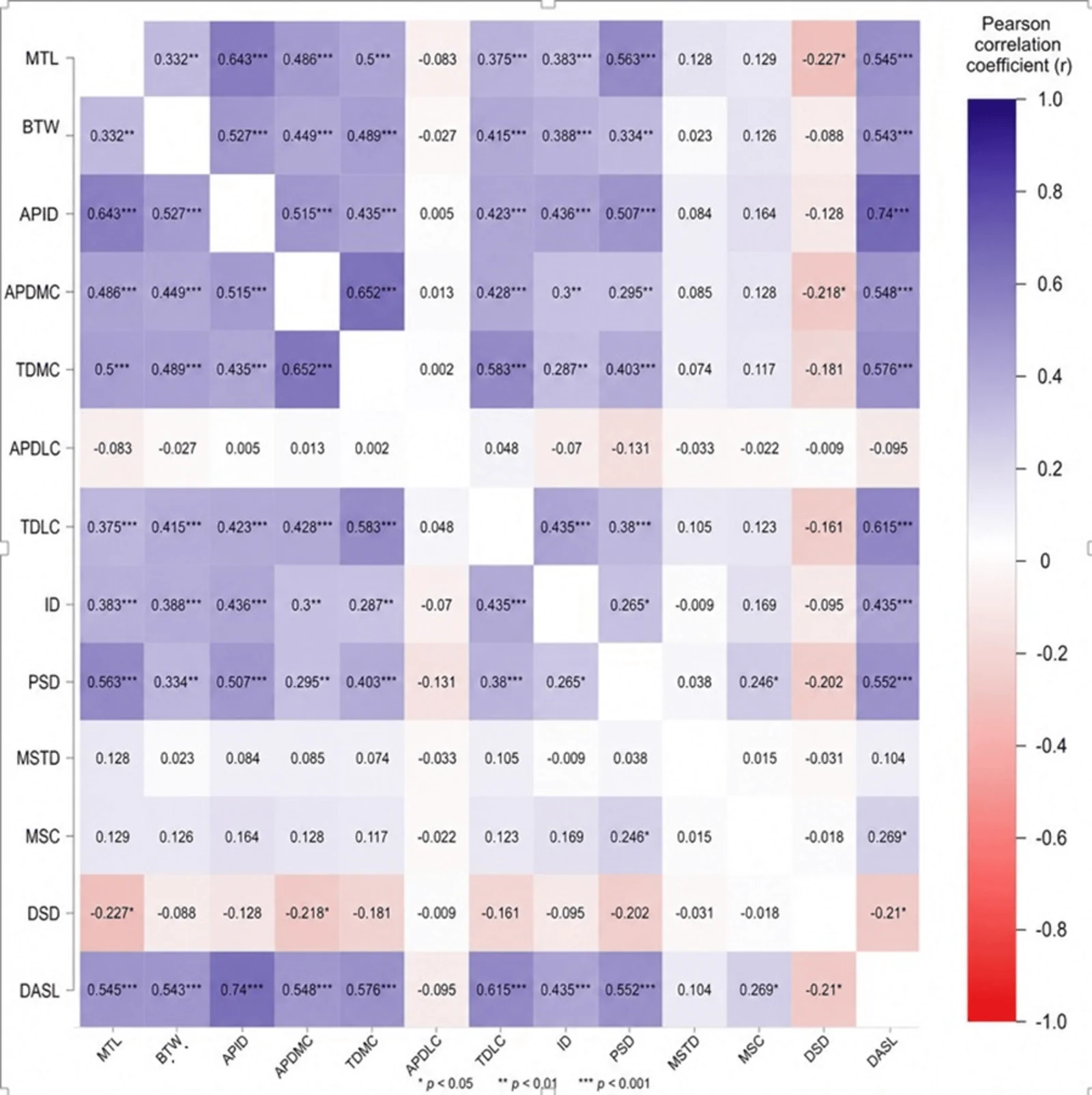
Heat map showing Pearson correlation coefficients (r) among the studied variables. * p < 0.05, ** p < 0.01, and *** p < 0.001. MTL: maximum tibial length; APDMC: anterior-posterior diameter of the medial condyle; BTW: bicondylar tibial width; APID: anterior-posterior intercondylar diameter; TDMC: transverse diameter of the medial condyle; APDLC: anterior-posterior distance of the lateral condyle; TDLC: transverse diameter of the lateral condyle; ID: intercondylar distance; PSD: proximal shaft diameter; MTSD: midshaft transverse diameter; MSC: mid-shaft circumference; DSD: distal shaft diameter; DASL: distal articular surface length.

## Discussion

The present study provides population-specific tibial morphometric data and demonstrates significant associations between several proximal tibial parameters and MTL in a North Indian population. The strong positive correlation of MTL with APID, along with the association between APID and DASL, suggests coordinated development of tibial dimensions and may reflect functional adaptation to weight-bearing and locomotion [4,10,11].

Males showed significantly greater MTL and tibial breadths than females, confirming the sexual dimorphism reported in previous osteometric studies [2,12,13]. The better performance of regression models in males may reflect lower variability in tibial dimensions and emphasizes the value of sex-specific equations for forensic estimation [14].

Greater variability and non-normality in shaft parameters, particularly mid-shaft transverse and distal shaft diameters, may partly reflect age-related remodeling and degenerative changes in this relatively older sample [15,16]. These alterations could explain the weaker or negative associations of some diaphyseal measurements with MTL, suggesting that proximal articular dimensions may provide more stable predictors of tibial length in older individuals.

The regression equations developed here may assist forensic estimation when only fragmentary tibial remains are available. Population-specific equations generally provide greater accuracy than generalized standards [17-19], making the present North Indian data particularly relevant given India's marked regional diversity.

The study is limited by its relatively small sample size, predominance of older individuals, and use of dry bones, which restricts extrapolation to younger populations and direct comparison with living anthropometric data. Larger, age-stratified studies incorporating radiological and in vivo measurements are warranted. Overall, the findings provide useful baseline data and support the application of proximal tibial morphometry for estimating MTL in the North Indian population.

## Conclusions

The study successfully derived population-specific regression models for estimating MTL from anthropometric measurements in the North Indian population. These models can be applied in forensic investigations and anthropological research to estimate stature accurately. However, further validation studies in diverse Indian subpopulations are recommended to enhance the generalizability of the findings.
